# Construction of a ceRNA-based lncRNA–mRNA network to identify functional lncRNAs in premature ovarian insufficiency

**DOI:** 10.3389/fgene.2022.956805

**Published:** 2022-10-13

**Authors:** Chao Luo, Jiakai Zhang, Le Bo, Lun Wei, Guangzhao Yang, Shasha Gao, Caiping Mao

**Affiliations:** ^1^ Reproductive Medicine Center, First Affiliated Hospital of Soochow University, Suzhou, Jiangsu, China; ^2^ Monash University, Caulfield East, Melbourne, VIC, Australia

**Keywords:** Premature ovarian insufficiency, long non-coding RNA, competing endogenous RNA, bioinformatics analysis, lncRNA–mRNA network

## Abstract

Premature ovarian insufficiency, characterized by ovarian infertility and low fertility, has become a significant problem in developed countries due to its propensity for late delivery. It has been described that the vital role of lncRNA in the development and progression of POI. The aim of this work was to create a POI-based lncRNA–mRNA network (POILMN) to recognize key lncRNAs. Overall, differently expressed mRNAs (DEGs) and differently expressed lncRNAs (DELs) were achieved by using the AnnoProbe and limma R packages. POI-based lncRNA–mRNA network (POILMN) construction was carried out using the tinyarray R package and hypergeometric distribution. To identify key lncRNAs, we used CentiScaPe plug-in Cytoscape as a screening tool. In total, 244 differentially expressed lncRNAs (DELs) and 288 differentially expressed mRNAs (DEGs) were obtained in this study. Also, 177 lncRNA/mRNA pairs (including 39 lncRNAs and 86 mRNAs) were selected using the hypergeometric test. Finally, we identified four lncRNA (HCP5, NUTM2A-AS1, GABPB1-IT1, and SMIM25) intersections by topological analysis between two centralities (degree and betweenness), and we explored their subnetwork GO and KEGG pathway enrichment analysis. Here, we have provided strong evidence for a relationship with apoptosis, DNA repair damage, and energy metabolism terms and pathways in the key lncRNAs in our POI-based lncRNA–mRNA network. In addition, we evaluated the localization information of genes related to POI and found that genes were more distributed on chromosomes 15, 16, 17, and 19. However, more experiments are needed to confirm the functional significance of such predicted lncRNA/mRNA. In conclusion, our study identified four long non-coding RNA molecules that may be relevant to the progress of premature ovarian insufficiency.

## Introduction

Premature ovarian insufficiency (POI) is a clinical condition exhibiting symptoms of ovarian hypofunction before the age of 40 (Touraine, 2020). It is characterized by menstrual disturbance (amenorrhea or oligomenorrhea) with raised gonadotropins (FSH>25 U/L) and fluctuating drop in estrogen levels, and is estimated to affect 1% of women (European Society for Human Reproduction and Embryology (ESHRE) Guideline Group on POI et al., 2016). Since the pathogenesis of the disease is still unclear, the lack of effective biomarkers and therapeutic targets poses a challenge for early diagnosis and treatment. Furthermore, China’s low fertility rate has also raised a serious question about fertility preservation in women of childbearing age with POI (Liang et al., 2019). Therefore, we must first identify the genes that play an important role in the POI process.

Long non-coding RNAs (lncRNAs) are broadly classified as transcripts longer than 200 nucleotides that are 5′ capped and polyadenylated like most mRNAs, yet this class of transcripts has limited coding potential (Klattenhoff et al., 2013). Because of the development of microarray and RNA-sequencing, we can explore key molecules in disease from multiple perspectives (Raza et al., 2022, 6). Emerging evidence indicates that lncRNAs play critical roles in various biological processes, such as cellular development, differentiation, imprinting control, immune response, and chromatin modification (Rinn et al., 2007; Amaral and Mattick, 2008; Dinger et al., 2008; Ponting et al., 2009; Lee, 2012). Conclusions drawn from different microarray analyses proved that many lncRNAs are unusually expressed in human granulosa cells; this means lncRNAs may be involved in the pathogenesis and progression of POI. For example, the decreased expression of HCP5 is directly related to the apoptosis of granulosa cells and DNA damage repair (Wang et al., 2020, 5). LncRNA DDGC was able to ameliorate the etoposide-induced DNA damage and apoptosis *in vivo* (Li et al., 2021b). Additionally, PVT1 ameliorates granulosa cell apoptosis by promoting SCP4-mediated Foxo3a dephosphorylation (Wang et al., 2021, 1). Therefore, only a few lncRNAs have been further elaborated so far, while the discoveries and confirmations of the vast majority are still an enigma. The competitive endogenous RNA hypothesis is mainly composed of mRNAs and lncRNAs who both share miRNA recognition elements and can compete with each other to occupy miRNAs (Salmena et al., 2011). The ceRNA mechanism in a variety of cancers and gynecological illnesses has been reported; at the same time, they also isolated some putative ceRNA networks, such as epithelial ovarian cancer (Zhao et al., 2019), polycystic ovary syndrome (Ma et al., 2021), and implantation failure (Feng et al., 2018). With insights into the mechanism of ceRNA, in this study, we aim to design a POI-based lncRNA–mRNA network (POILMN) to label key lncRNAs and explore gene location information and functional enrichment that may also point to directions for future research.

## Materials and methods

### Microarray data

In order to obtain the microarray analysis, the public database Gene Expression Omnibus (GEO) (https://www.ncbi.nlm.nih.gov/geo/) was searched using keywords such as “premature ovarian insufficiency,” “POI,” “granulosa cells,” and “Homo sapiens.” The GSE135697 dataset was selected for further study. The GSE135697 dataset (platform: GPL21096, Agilent-045997 Arraystar human lncRNA microarray V3) included lncRNA and mRNA expression profiles consisting of 10 POI samples and 10 control samples. The profiling construction and test of the datasets were authorized by the local research ethics committee.

### Differential gene expression analysis and probe reannotation

Preprocessed data were acquired from GEO using the R package “GEOquery”. After getting the expression matrix, and subsequently, according to the annotation profile recorded in the “AnnoProbe” package (version 0.1.6), probesets were annotated to filter out the duplicate and unannotated probes and then separated into two categories: protein-coding dataset and lncRNA dataset. Log-transformed intensities were quantile normalized using the “normalizeBetweenArrays” function in the “limma” package of R. Then, the limma package was used to identify differentially expressed mRNAs and lncRNAs, respectively. The *p*-value was adjusted using the Benjamini–Hochberg method. Unless stated otherwise, “differentially expressed” (DE) mRNAs and lncRNA were defined as FDR <0.05 and log_2_|fold change| > 1.

### The location distribution of differential genes on chromosomes

There is growing evidence that POI was judged to be related to the genetically heterogeneous disorder. The chromosomal location and the starting and ending positions were assessed using the R package “AnnoProbe” (version 0.1.6). The R library “RIdeogram” was used to visualize data along the chromosomes of differentially expressed mRNAs and lncRNAs.

### LncRNA–miRNA and miRNA–mRNA interaction data and construction of the POILM network

Starbase v3.0 (http://starbase.sysu.edu.cn) was used to extract lncRNA–miRNA associations from HITS-CLIP and PAR-CLIP experiments. Sequence-predicted miRNA–mRNA pairs were obtained from miRTarBase (https://mirtarbase.cuhk.edu.cn/). The processed relation pairs are selected as the background relation pairs for the hypergeometric distribution.

To construct the POILMN, the DELs and DEGs were substituted in the background network *via* the R package tinyarray. Then, the lncRNA–miRNA–mRNA network was filtered *via* a hypergeometric test with *p* < 0.01, and counts >3 denote the counts of the number of miRNAs shared between lncRNA and mRNA. The value of *p* was calculated as

$$P=1-\overset{r-1}{\underset{i=0}{\sum }}\frac{\left(\frac{t}{i}\right)\left(\frac{m-t}{n-i}\right)}{\left(\frac{m}{n}\right)}.$$

In the formula of hypergeometric distribution, m is the total miRNA number in the miRTarBase database, n represents the number of miRNAs interacting with a lncRNA, t is the number of miRNAs interacting with an mRNA, and r indicates the quantities of miRNAs united between the lncRNA/mRNA pair.

### Functional and pathway enrichment analysis

Gene Ontology (GO) and Kyoto Encyclopedia of Genes and Genomes (KEGG) pathway analysis of the DEGs sifted out in the POILMN and lncRNA subnetwork was performed and visualized using the R package clusterProfiler (V4.2.2) (Wu et al., 2021). KEGG pathways and Gene Ontology (GO) terms were considered statistically significant using *p* < 0.05 as the cut-off value. The GO enrichment analysis consists of three components molecular functions (MFs), biological processes (BPs), and cellular components (CCs).

### Topological analysis and selection of key lncRNAs

To explore the central nodes of the POILMN network, we performed a topological analysis of DELs and DEGs and calculated using the CentiScaPe plug-in Cytoscape. We focused on two main topological parameters: “degree” and “betweenness.” Retain the topped-eight lncRNA of each parameterization and those overlapped were chosen as hub genes for the follow-up stage.

### Construction of ceRNA sub-networks

Through the implementation of the previous approach using a hypergeometric test, we obtained all the key lncRNAs and their adjacent mRNA neighbors. At the same time, we also get the overlapped miRNAs that they commonly shared. Ultimately, based on ceRNA theory, we imported a triple network into Cytoscape software to visualize it.

### Subcellular localization analysis

To investigate the intracellular localization of key lncRNAs in topological analysis, we used a web-based public platform lncLocator, for prediction. Target lncRNA sequences were downloaded from NCBI in nucleotide FASTA format. Bar plotting was performed using R with the ggplot2 (V3.3.5) package.

## Results

### Differentially expressed lncRNAs and mRNAs

**FIGURE 1:**
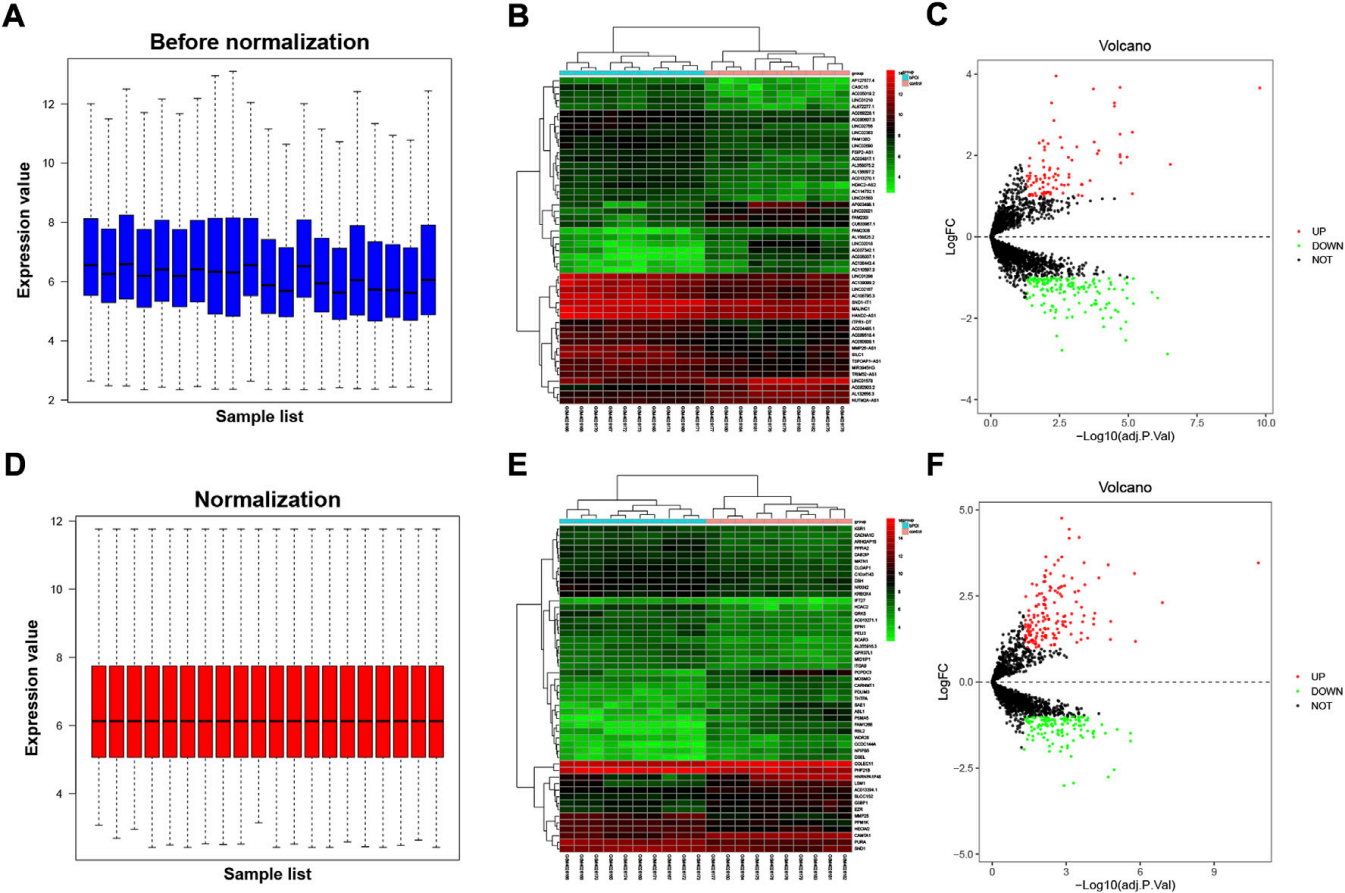
Differentially expressed lncRNAs and mRNAs from granulosa cells in patients with premature ovarian insufficiency (POI). The dataset was split into the lncRNA dataset and protein-coding dataset. Microarray data of dataset, **(A)** prior to normalization and **(B)** following normalization. **(C)** Heatmap of top 50 DELs (sorted by adjusted *p*-value); **(D)** heatmap of top 50 DEGs (sorted by adjusted *p*-value). Volcano plot showing differentially expressed lncRNAs **(E)** and mRNAs **(F)** after being screened by FDR <0.05 and log2|fold change| > 1. Red dots and green dots referred to the upregulated and downregulated genes, respectively.

**FIGURE 2:**
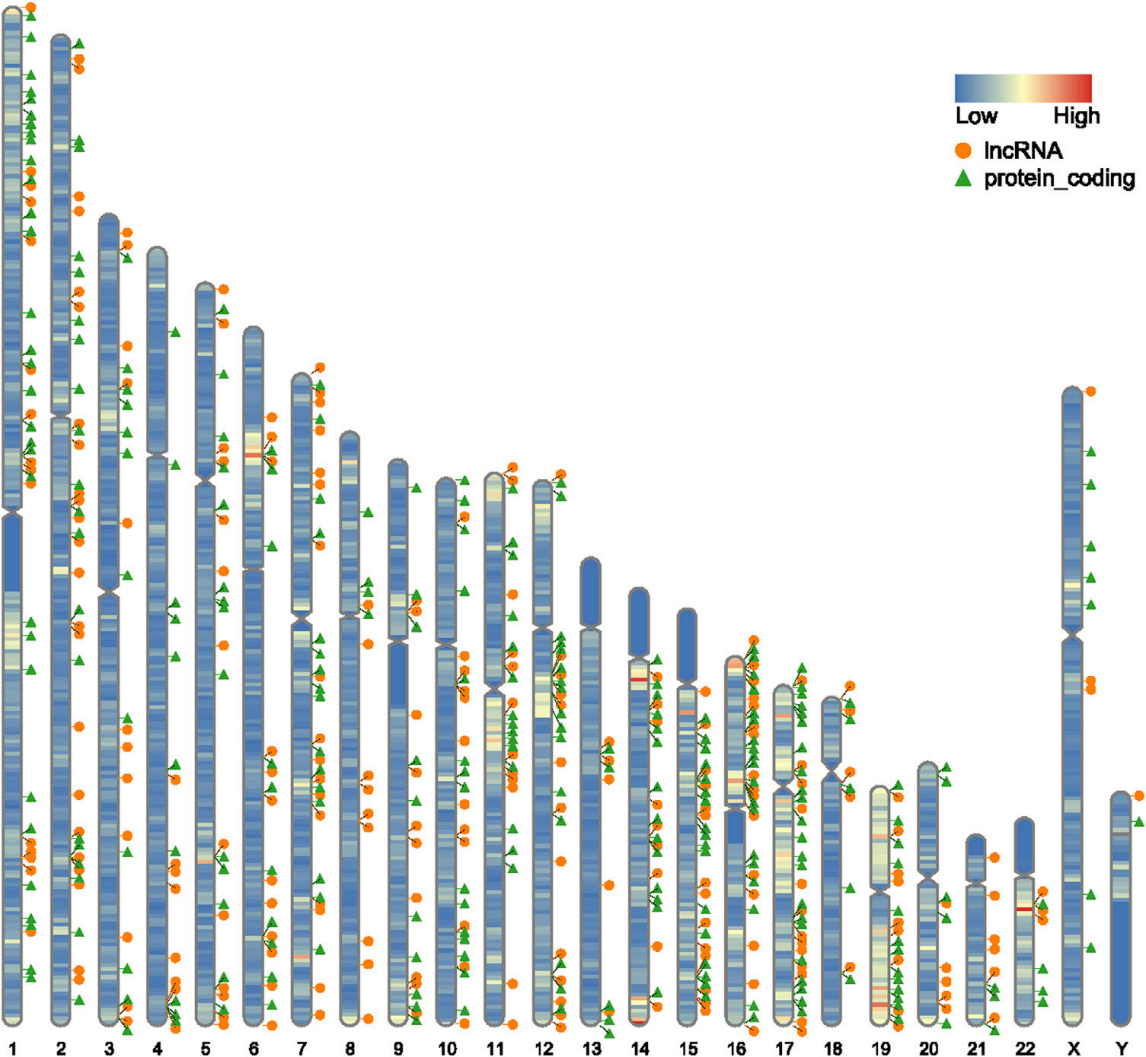
Schematic diagram of the distribution of differentially expressed mRNA and lncRNA on chromosomes, orange circles represent lncRNAs, green triangles represent protein-coding, and the color on each chromosome represents gene density.

Expression estimates were further normalized using quantile normalization; box plots show mean expression level differences before and after normalization in Figures 1A,B. In GSE135697, 244 DELs were identified with log2|fold change| > 1 and *p* < 0.05, including 93 upregulated and 151 downregulated, in the POI granulosa cells in the test compared to the control, as shown in the volcano plot (Figure 1E); At the same time, there were 288 DEGs, with 160 upregulated and 128 downregulated (Figure 1F). The list of 288 DEGs and 244 DELs is shown in Supplementary Table S1. Several genome-wide specific genomic features were revealed at the chromosomal level, as shown in Figure 2.

### Construction of the POI-related lncRNA–mRNA network

**FIGURE 3:**
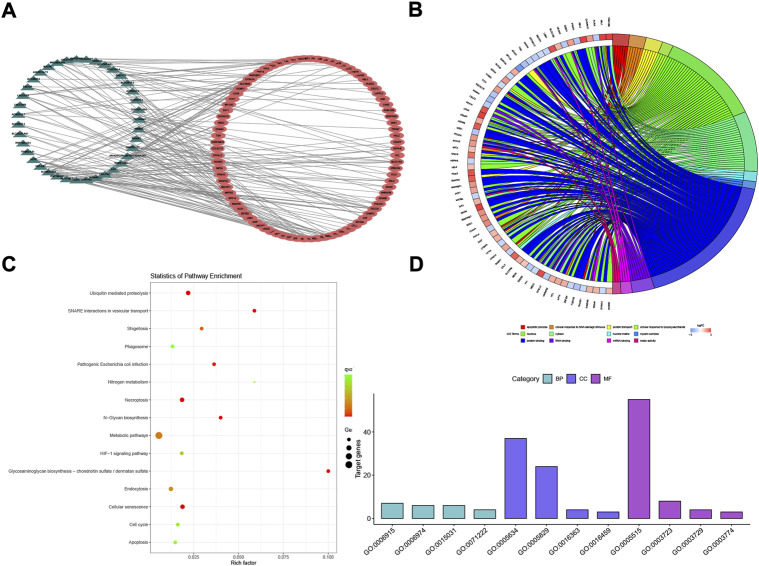
POI-based lncRNA–mRNA network (POILMN) and functional enrichment analysis of mRNAs. The dark green triangles represent lncRNA, and the red cycles represent mRNAs. There were 39 lncRNA nodes, 86 mRNA nodes, and 177 edges in the network **(A)**. Distribution of integrated DEGs in premature ovarian insufficiency for different GO-enriched functions **(B)**. KEGG pathway enrichment analysis of the integrated DEGs **(C)**. The GO enrichment bar chart of DEGs presents the number of DEGs enriched in biological processes, cellular components, and molecular functions **(D)**.

After the stringent filtering, we ended up with 63,698 lncRNA–miRNA pairs (including 642 miRNAs and 3,788 lncRNAs) and 502,653 miRNA–mRNA interaction pairs (including 15,064 mRNA and 2,599 miRNAs). Total DELs/DEMs were matched into those two interaction pairs, and then 177 lncRNA/mRNA pairs (including 39 lncRNAs and 86 mRNAs) were selected using the hypergeometric test with *p* < 0.01 and counts >3 in Figure 3A. The lncRNA/mRNA pairs are shown in Supplementary Table S2.

### Functional enrichment analysis of the differentially expressed mRNAs in the ceRNA network

To obtain a better understanding of the role of the DEGs in the lncRNA–mRNA ceRNA network, we performed GO classification and KEGG pathway analysis. Results showed that 16 GO terms (*p* < 0.05) were significantly enriched in the GO analysis. The top four GO terms in BP, MF, and CC are listed in Figure 3D. Biological process (BP) analysis showed that the associated mRNAs were “cellular response to DNA damage stimulus”. Chen et al. (Wang et al., 2020, 5) have proved that reduced expression of long non-coding RNA HCP5 in POI modulates the repair of MSH5 transcription and DNA damage interacting with YB1, leading to GC dysfunction, providing potent evidence for POI pathogenesis in the cellular response to DNA damage stimulus. On the MF dimension, the top four terms associated with gene counts were protein binding, RNA binding, mRNA binding, and motor activity. The KEGG pathways showing the most significant enrichment were glycosaminoglycan biosynthesis—chondroitin sulfate/dermatan sulfate, SNARE interactions in vesicular transport, ubiquitin-mediated proteolysis, and cellular senescence, as shown in Figure 3C. Herein, these GO terms and KEGG pathways may shed new light on POI pathogenesis and prognosis.

### Topological characteristics of the POI-based lncRNA–mRNA network and locations of key lncRNAs

The topological features of the POILMN, including degrees and betweenness, were chosen to forecast the biological functions of the lncRNAs in POILMN. Then, the top eight sorted genes in the POILMN with the highest value were extracted. The intersection of lncRNAs was screened at the degree and betweenness parameter, and four lncRNAs, that is, NUTM2A-AS1, HCP5, SMIM25, and GABPB1-IT1, were jointly identified by the intersection between two features.

**FIGURE 4:**
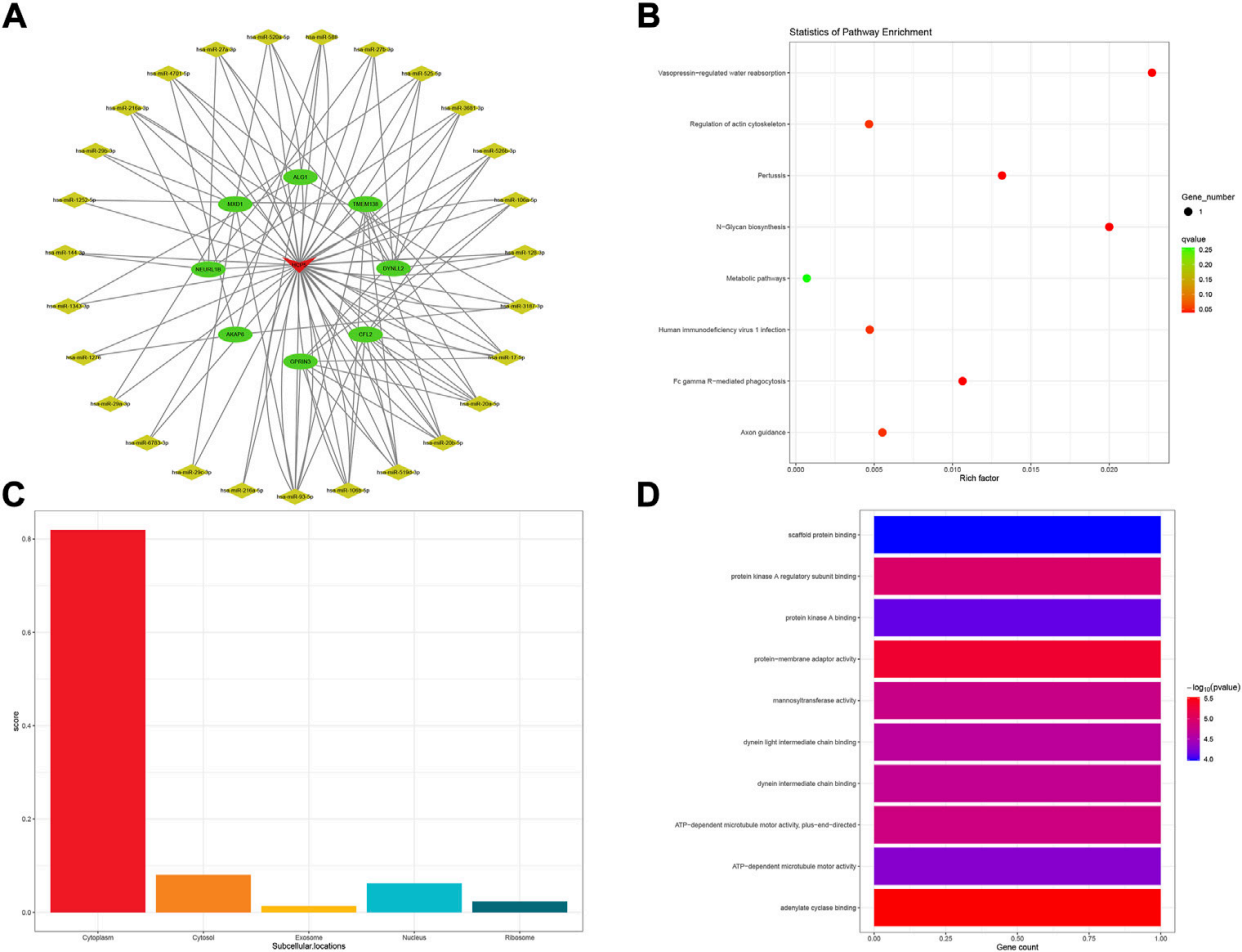
HCP5-related ceRNA subnetwork analysis. ceRNA network of HCP5 **(A)**. KEGG pathways enriched in HCP5 **(B)**. Subcellular location analysis for HCP5 **(C)**. GO biological process enrichment results for HCP5 **(D)**.

Comprehensive rating had eight top results for HCP5, NUTM2A-AS1, SMIM25, and GABPB1-IT1, respectively. For HCP5, a total of eight mRNAs and 36 miRNAs were composed of the subnetwork of the POILMN in Figure 4A. A total of 8 KEGG pathways were significantly enriched (*p* < 0.05) in the KEGG pathways analysis, as shown in Figure 4B. Most of HCP5 is localized in the cytoplasm (score = 0.819), and only a small part of it is localized to the nucleus, ribosome, cytosol, and exosome (Figure 4C). GO classification with *p* ≤ 0.05 adjusted by Benjamini–Hochberg found ten enriched GO terms for molecular function, as shown in Figure 4D.

**FIGURE 5:**
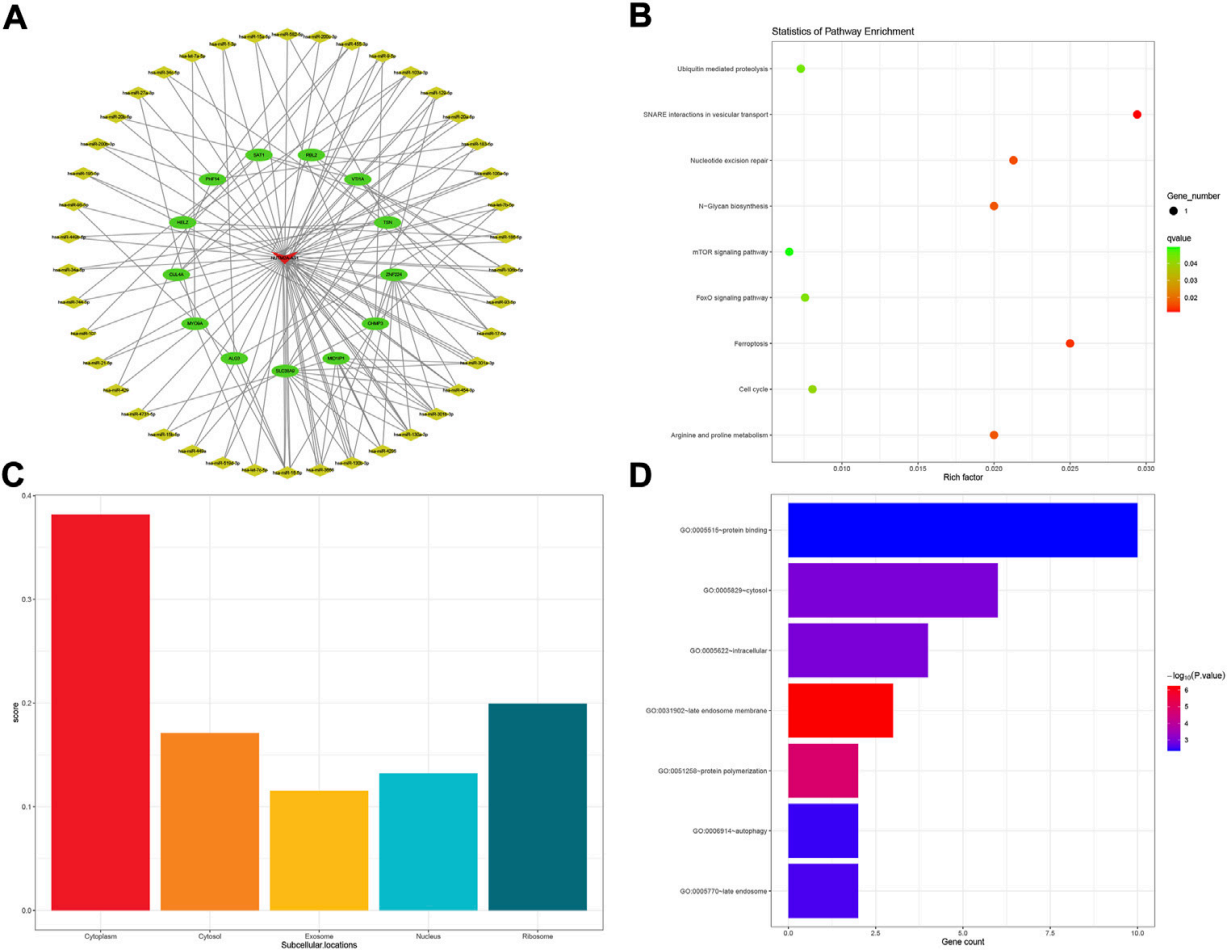
NUTM2A-AS1-related ceRNA subnetwork analysis. ceRNA network of NUTM2A-AS1 **(A)**. KEGG pathways enriched in NUTM2A-AS1 **(B)**. Subcellular location analysis for NUTM2A-AS1 **(C)**. GO biological process enrichment results for NUTM2A-AS1 **(D)**.

As for the NUTM2A-AS1 subnetwork, 13 mRNA and 43 miRNA comprised the subnetwork (Figure 5A). KEGG pathways analysis showed that nine pathways were enriched in the process, as shown in Figure 5B. Lnclocator analysis revealed that NUTM2A-AS1 is mainly distributed in the cytoplasm and is being distributed in the nucleus, ribosome, cytosol, and exosome at the same time (Figure 5C). In the GO enrichment analysis for the NUTM2A-AS1 subnetwork, we got seven terms, two terms in BP, one in MF, and four in CC (Figure 5D).

**FIGURE 6:**
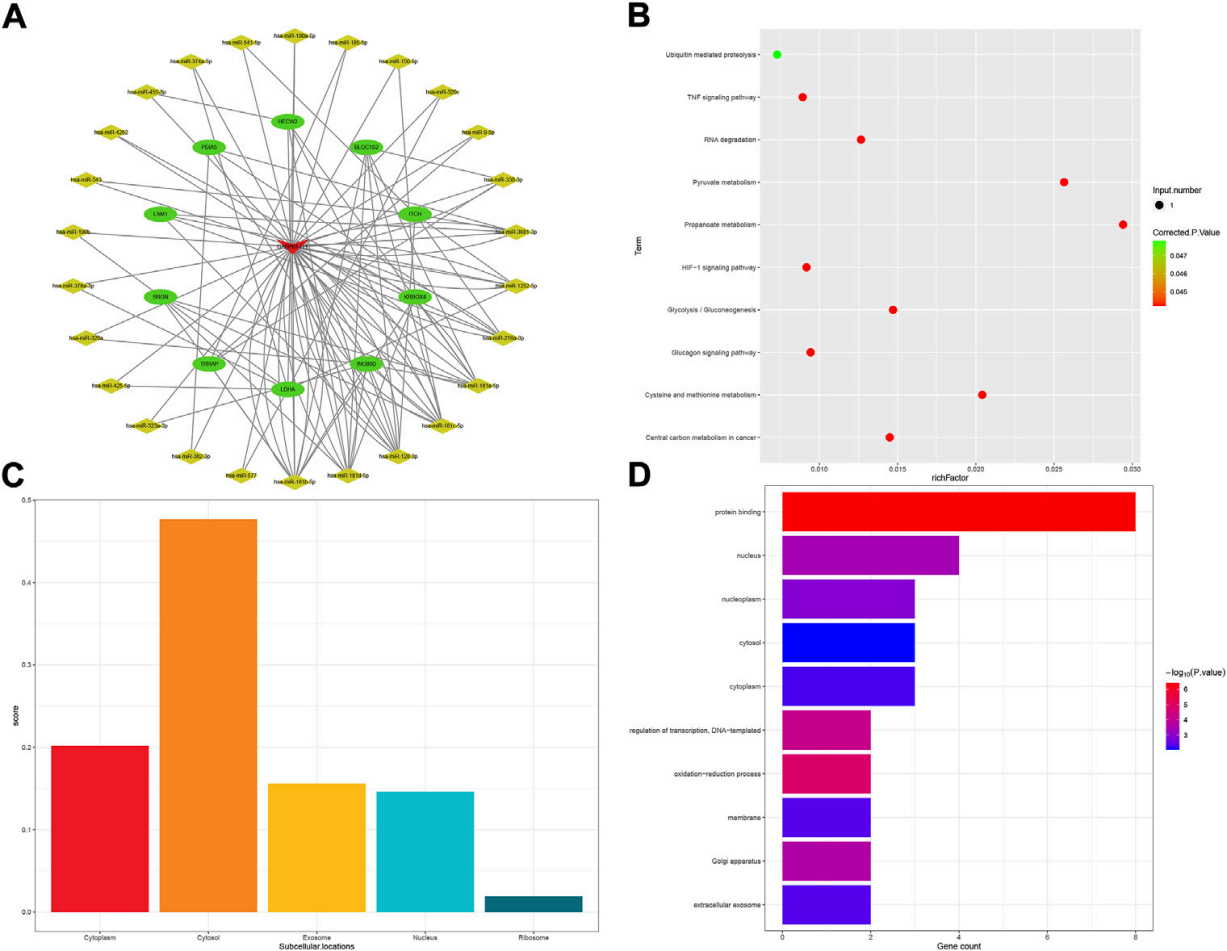
GABPB1-IT1-related ceRNA subnetwork analysis. ceRNA network of GABPB1-IT1 **(A)**. KEGG pathways enriched in GABPB1-IT1 **(B)**. Subcellular location analysis for GABPB1-IT1 **(C)**. GO biological process enrichment results for GABPB1-IT1 **(D)**.

The GABPB1-IT1 subnetwork was made up of 10 mRNA and 28 miRNA in all (Figure 6A). In conducting KEGG analysis, 11 KEGG pathways were significant among 14 KEGG pathways in total (Figure 6B). GABPB1-IT1 was mostly localized to the cytosol (score = 0.477) (Figure 6C). In contrast, GO term analysis revealed that target genes were involved in 109 GO terms (*p* < 0.05). Only the top ten GO biological process terms are shown in Figure 6D.

**FIGURE 7:**
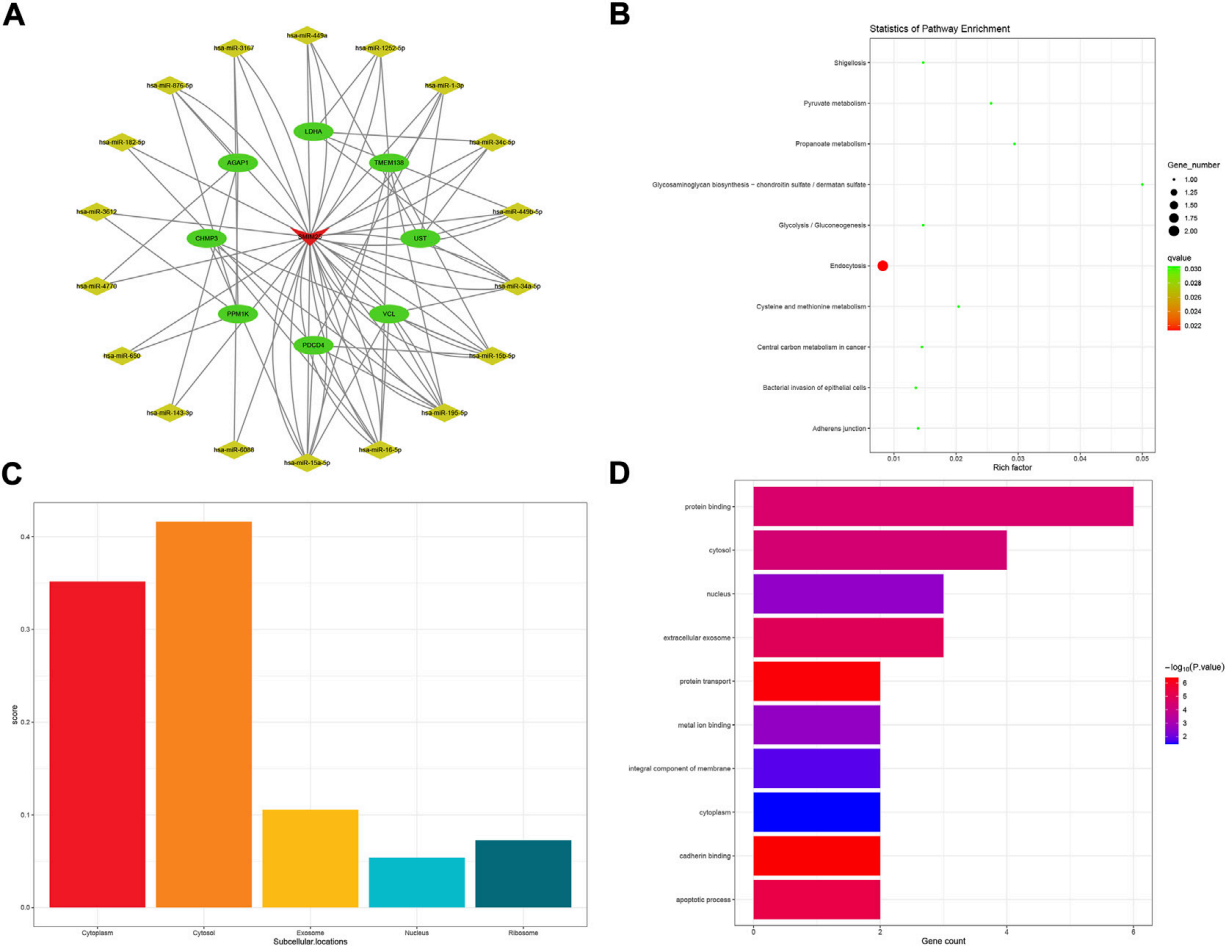
SMIM25-related ceRNA subnetwork analysis. ceRNA network of SMIM25 **(A)**. KEGG pathways enriched in SMIM25 **(B)**. Subcellular location analysis for SMIM25 **(C)**. GO biological process enrichment results for SMIM25 **(D)**.

The SMIM25 subnetwork was composed of eight mRNA and 19 miRNA (Figure 7A). The enrichment analysis of KEGG pathways included 18 KEGG pathways; among them, the top 10 pathways are visualized in Figure 7B. SMIM25 was mainly localized to the cytoplasm (score = 0.352) and cytosol (score = 0.416), as shown in Figure 7C. Upon GO classification, 2 CC and 8 MF terms were obtained to be enriched in SMIM25 (Figure 7D).

## Discussion

POIs are a common disorder of follicular development characterized by hypertrophy of the initial follicle, slowed growth of antral follicles, and selection of the dominant follicle. In our study, we sought to identify these lncRNAs by examining data from POI microchips and performing analyses to determine their potential roles in POILMN.

Chromosomal abnormalities have long been recognized as a frequent cause of POI. We restored the differentially expressed mRNAs and lncRNAs to their chromosomal locations and found that they were not only associated with autosomes but also with sex chromosomes. At the same time, it was found that there are a large number of distributions on the long arm of chromosome 15, the short arm of chromosome 16, and chromosomes 17 and 19.

The lncRNA/miRNA and mRNA/miRNA pairs were obtained from the starBase and miRTarBase databases, respectively, and the lncRNA–mRNA network was constructed by calculating the hypergeometric distribution using differentially expressed mRNAs and lncRNAs. The POI-based lncRNA–mRNA network (POILMN) was then extracted with 86 mRNA nodes, 39 lncRNA nodes, and 177 edges. Results were analyzed to determine putative functional POI biomarkers.

For the GO term enrichment analyses of DEGs in the POILMN, we observed a large proportion in the number of counts in the category of biological process (BP), such as “apoptotic process,” “cellular response to DNA damage stimulus,” and “protein transport.” Considering of its GC-related diseases, some previous studies confirmed that decreasing apoptosis of granulosa cells could improve the function of the ovary in POI mice (Huang et al., 2019, 1; Ling et al., 2019; Wang et al., 2019). A study found that low expression of lncRNA HCP5 in granulosa cells from POI patients interfered with GC DNA damage healing, promoting apoptosis of GCs, and mediated the translocation of YB1 protein to the GC nucleus (Wang et al., 2020, 5). As for the molecular functions (MFs) and cellular components (CCs) categories, the primary term was “nucleus” and “protein binding.”

Intriguingly, the most enriched KEGG pathway was “metabolic pathways.” Aside from lower serum AMH levels and higher FSH levels, we also retrieved some metabolism-related case-control studies of POI. These association studies pointed out that serum concentrations of total cholesterol (TC), high-density lipoprotein cholesterol (HDL-C), and low-density lipoprotein cholesterol (LDL-C) were significantly higher in the POI group compared with a healthy matched control group (Ates et al., 2014; Podfigurna et al., 2018). Another cross-sectional case-control study points out women with POI were more likely to exhibit increased serum levels of TG (β, 0.155; 95% CI, 0.086, 0.223) and glucose (0.067; 0.052, 0.083), decreased levels of HDL-C (−0.087; −0.123, −0.051), LDL-C (−0.047; −0.091, −0.003) and uric acid (−0.053; −0.090, −0.015), and impaired kidney function (urea [0.070; 0.033, 0.107]; creatinine [0.277; 0.256, 0.299]; and eGFR [−0.234; −0.252, −0.216]) compared with controls after adjusting for age and BMI (Huang et al., 2021). In conclusion, a higher risk of metabolic syndrome was associated with POI. It also reminds us to adopt lifetime management of metabolic abnormalities that are needed in the early diagnosis of POI.

Several carbohydrate metabolism-related pathways were also enriched, including “glycosaminoglycan biosynthesis − chondroitin sulfate/dermatan sulfate” and “N-glycan biosynthesis.” Some studies have reported that glycosaminoglycan chondroitin-4-sulfate may play a role in altering gonadotrophin-stimulated and basal progesterone secretion in follicles during the differentiation of granulosa cells (Ledwitz-Rigby et al., 1987). “HIF-1 signaling pathway” was another KEGG term enriched in our POILMN. According to previous research studies, ROS accumulation induces oxidative damage to ovarian GCs, hence prompting the onset of follicular atresia and relevant anovulatory disorders, such as POI (Agarwal et al., 2012). More importantly, ROS are involved in the hypoxia response through a mechanism that stabilizes hypoxia-inducible factor 1 (Chandel et al., 2000).

We performed a topology analysis of lncRNAs, calculated topological parameters (betweenness and degree), and identified four candidate lncRNAs (HCP5, NUTM2A-AS1, GABPB1-IT1, and SMIM25) that may potentially affect POI susceptibility.

The KEGG pathway of “vasopressin-regulated water reabsorption” was enriched in the ceRNA subnetwork of HCP5. Studies have shown that exposure to high doses of PFOA increases the risk of premature ovarian insufficiency by reducing pituitary expression in the suprachiasmatic nucleus (SCN); this implies a possible connection between POI and vasopressin in some way (Zhang et al., 2020). In addition, we also found that “N-glycan biosynthesis” and “metabolic pathways” exist in the enrichment of pathways; the same pathways were also expounding in the DEGs enrichment as before. Several energy metabolism-related GO terms, including “ATP-dependent microtubule motor activity, plus-end-directed,” “ATP-dependent microtubule motor activity,” and “adenylate cyclase binding” were enriched. Energy metabolism may play a central role in many physiological and pathological processes when HCP5 is activated and functioning. As for “protein kinase A regulatory subunit binding” and “protein kinase A binding” terms, although a few hundreds of protein kinases regulate key processes in human cells, protein kinases play a pivotal role in health and disease. This study demonstrates that protein kinase A appears to be an important upstream kinase sufficient to initiate complex intracellular signaling pathways and gene expression profiles associated with GC differentiation (Puri et al., 2016).

The KEGG “mTOR signaling pathway” and GO term “autophagy” were enriched in the ceRNA subnetwork of NUTM2A-AS1. Autophagy is an evolutionarily conserved cellular process controlled through a set of essential autophagy genes (Atgs). As an important player in autophagy, mTOR is essential for autophagosome formation and necessary for the closure of isolation membranes of autophagosomes. The activated mTOR pathway stimulates the proliferation of granulosa cells (Kayampilly and Menon, 2007; Yu et al., 2011) and also participates in the regulation of ovarian steroidogenesis. In some cases, researchers unveiled a novel role for mTOR signaling in the maintenance of granulosa cellular homeostasis by regulating autophagy at the transcriptional level (Yin et al., 2020).

For GO term enrichment analysis, the “oxidation-reduction process” was enriched in the ceRNA sub-network of GABPB1-IT1. Oxidative stress-induced granulosa cell (GC) death represents a common reason for follicular atresia, which can cause amenorrhea beforehand. As for the KEGG pathway “glycolysis/gluconeogenesis,” a recent study showed that the energy stress-induced lncRNA ZNF674-AS1 regulates GC proliferation and glycolysis, possibly contributing to follicular dysfunction (Li et al., 2021a).

There are also glucose metabolism pathways enriched in SMIM25 KEGG analysis, such as “glycolysis/gluconeogenesis” and “propanoate metabolism,” that further emphasize the relationship between POI and energy metabolism. By comparing the analysis results of GO enrichments, we found that the “extracellular exosome” term was enriched. Exosomes are extracellular vesicles that mediate cellular communication in health and disease. It has also been shown that exosomes contain messenger RNAs (mRNAs) and microRNAs (miRNAs), which can be delivered unidirectionally and functionally between cells (Ratajczak et al., 2006; Valadi et al., 2007). Recent studies have shown that MSC-derived exosomes supplementation can restore ovarian function in premature ovarian insufficiency (Sun et al., 2019; Ding et al., 2020, 7; Yang et al., 2020).

In brief, our research provided a global view of ceRNA, lncRNA, and mRNA with potential implications for the onset and development of POI. Nevertheless, further longitudinal studies are necessary to extend and explore these potential lncRNAs.

## Data Availability

The original contributions presented in the study are included in the article/Supplementary Material; further inquiries can be directed to the corresponding author.
